# Hepatitis C virus itself is a causal risk factor for chronic kidney disease beyond traditional risk factors: a 6-year nationwide cohort study across Taiwan

**DOI:** 10.1186/1471-2369-14-187

**Published:** 2013-09-06

**Authors:** Yi-Chun Chen, Wen-Yen Chiou, Shih-Kai Hung, Yu-Chieh Su, Shang-Jyh Hwang

**Affiliations:** 1 Divisions of Nephrology, Department of Internal Medicine, Buddhist Dalin Tzu Chi General Hospital, Chiayi and School of Medicine, Tzu Chi University, Hualien, Taiwan; 2 Department of Radiation Oncology, Buddhist Dalin Tzu Chi General Hospital, Chiayi, and School of Medicine, Tzu Chi University, Hualien, Taiwan; 3 Divisions of Hematology-Oncology, Department of Internal Medicine, Buddhist Dalin Tzu Chi General Hospital, Chiayi and School of Medicine, Tzu Chi University, Hualien, Taiwan; 4 Division of Nephrology, Department of Internal Medicine, Kaohsiung Medical University Hospital, Kaohsiung, Taiwan

**Keywords:** Hepatitis C virus, Chronic kidney disease, Taiwan national health insurance research database, Cohort study

## Abstract

**Background:**

Hepatitis C virus (HCV) infection and chronic kidney disease (CKD) have high prevalences in Taiwan and worldwide, but the role of HCV infection in causing CKD remains uncertain. This cohort study aimed to explore this association.

**Methods:**

This nationwide cohort study examined the association of HCV with CKD by analysis of sampled claims data from Taiwan National Health Insurance Research Database from 1998 to 2004. ICD-9 diagnosis codes were used to identify diseases. We extracted data of 3182 subjects who had newly identified HCV infection and no traditional CKD risk factors and data of randomly selected 12728 matched HCV-uninfected control subjects. Each subject was tracked for 6 years from the index date to identify incident CKD cases. Cox proportional hazard regression was used to determine the risk of CKD in the HCV-infected and control groups.

**Results:**

The mean follow-up durations were 5.88 years and 5.92 years for the HCV-infected and control groups, respectively. Among the sample of 15910 subjects, 251 subjects (1.6%) developed CKD during the 6-year follow-up period, 64 subjects (2.0%) from the HCV-infected group and 187 subjects (1.5%) from the control group. The incidence rate of CKD was significantly higher in the HCV-infected group than in the control group (3.42 *vs.* 2.48 per 1000 person-years, *p* = 0.02). Multivariate analysis indicated that the HCV-infected group had significantly greater risk for CKD (adjusted hazard ratio: 1.75, 95% CI: 1.25-2.43, *p* = 0.0009). This relationship also held for a comparison of HCV-infected and HCV-uninfected subjects who were younger than 70 years and had none of traditional CKD risk factors.

**Conclusions:**

HCV infection is associated with increased risk for CKD beyond the well-known traditional CKD risk factors. HCV patients should be informed of their increased risk for development of CKD and should be more closely monitored.

## Background

Chronic kidney disease (CKD) is an increasing public health problem worldwide. The well-recognized risk factors for CKD are advanced age [1], diabetes [1], hypertension [1], hyperlipidemia [1], coronary heart disease [2], and cirrhosis [2]. Infectious disease is a recently identified and under-recognized risk factor for CKD [3].

Hepatitis C has a high prevalence and affects about 170 million people worldwide. The hepatitis C virus (HCV) mainly causes liver damage, but is also associated with extra-hepatic diseases, including various types of glomerulonephritis [4], even in the absence of cirrhosis [5]. In addition, HCV is more common in CKD patients who are not yet on dialysis than in the general population [6]. Previous research indicated that HCV infection leads to a rapid decline in the renal function of patients with diabetic nephropathy [7] and of HCV-infected patients with cirrhosis who terminated interferon therapy [8]. Collectively, these studies suggest that HCV infection has an adverse impact on renal function.

However, there are conflicting results regarding the effect of HCV on renal function. Two cohort studies in America reported positive associations of HCV and CKD in the presence of cirrhosis [9] or ESRD [10] in a population of veterans, but two other studies of an urban hospital population and a private health insurance population reported no association [2,11]. These studies had adequate sample sizes, but short follow-up periods (<4 years) and a predominance of males, so the findings may not apply to the general population. The results of two other American [12,13] and two Taiwanese cross-sectional studies [14,15] were also inconsistent. However, another Taiwanese cohort study [16] indicated a positive association of HCV with ESRD. A meta-analysis of these observational studies indicated no association of HCV and CKD [17].

Molecular studies have shown that the HCV core protein directly inhibits insulin signalling and increases oxidative stress, which can exacerbate insulin resistance [18] and potentially lead to metabolic syndrome and advanced cirrhosis [19]. Insulin resistance and oxidative stress can also lead to endothelial dysfunction and is implicated in the progression of CKD [20]. These molecular studies provide a biological basis for the hypothesis that HCV infection increases the risk for CKD.

There is a significant and increasing burden of CKD and HCV infection in Taiwan, so Taiwan provides an ideal setting for a study of the relationship of these two diseases. The HCV population has a high comorbidity burden [21], and some of these comorbidities are well-known CKD risk factors, such as diabetes, hypertension, hyperlipidemia, coronary heart disease, and liver cirrhosis. A previous cohort study [2], based on a mean follow-up time of 25.3 months, reported no relationship of HCV and CKD. However, a cross-sectional retrospective analysis [22] reported that the mean time for development of CKD in the HCV-infected patients was 74 months. The purpose of the present cohort study was to examine the causal relationship of HCV and CKD over the course of 6 years, based on the review of the Taiwan National Health Insurance Research Database (NHIRD) and exclusion of HCV-infected subjects with well-known CKD risk factors.

## Methods

### Data source

This study used the NHIRD, released by the Taiwan National Health Research Institute (NHRI) and available to all researchers in Taiwan. The NHIRD has been widely used for epidemiological research [1,16,23-30], including studies of CKD [1,27] and hepatitis [16,23,24,28]. Taiwan initiated its National Health Insurance (NHI) program in March 1995. This is a compulsory-enrolment, single-payer system that finances healthcare for all Taiwanese citizens (~99% of the population) through a registry of board-certified specialists and contracted medical facilities. The NHIRD is a representative nationwide database that contains all original claims data for one million beneficiaries from 1996 to 2010, and is a random and systematic sample of the 25.68 million NHI enrolees. The NHRI reported no significant differences in age, gender, or healthcare costs for patients in the NHIRD and all NHI enrolees. Diagnostic coding by the NHI program in Taiwan is performed according to ICD-9 codes. Many previous studies have verified the accuracy of diagnoses in the NHIRD for major diseases such as diabetes, stroke, hepatitis, CKD, and ESRD [1,16,23,24,26-30]. This study was evaluated and approved by Taiwan’s National Health Research Institute Ethics Review Committee. All identifying personal information was removed from the secondary files before analysis, so the review board waived the requirement for written informed consent.

### Study population

**Figure 1:**
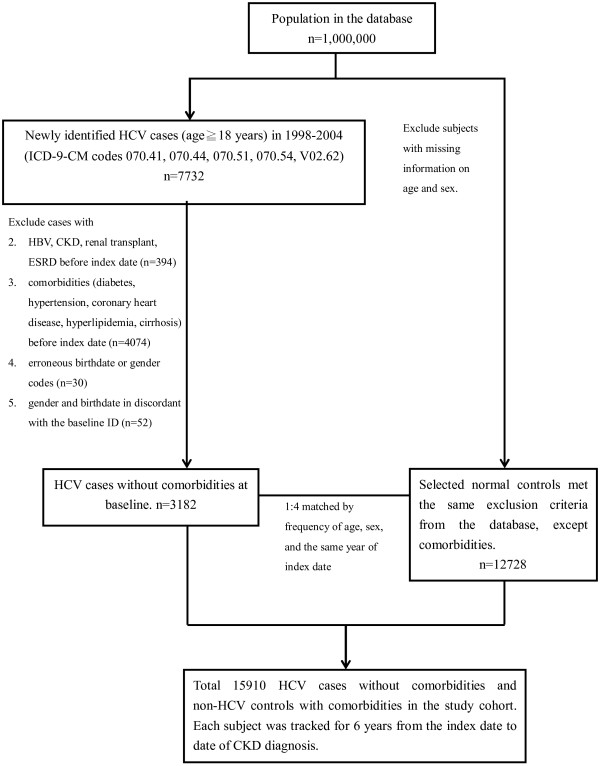
**Study flow chart.** Abbreviations: ESRD, end-stage renal disease; HBV, hepatitis B virus; HCV, hepatitis C virus; ICD-9-CM, International Classification of Diseases, Ninth Revision, Clinical Modification.

Figure 1 summarized the process used for selection of cases and controls in this retrospective cohort study. For the HCV-infected group, we identified all subjects 18 years or older who had a first-time diagnosis of HCV (ICD-9-CM codes 070.41, 070.44, 070.51, 070.54, V02.62) [16,23,24,28] over a 7-year period (January 1, 1998 to December 31, 2004) during an outpatient or hospitalization visit. The index date was defined as the date of the first diagnosis of HCV. Viral hepatitis is a significant public health problem in Taiwan, so the government closely monitors HCV infection and establishes detailed guidelines for diagnosis [24].

Subjects were excluded if they were diagnosed with HCV before 1998, diagnosed with HBV (ICD-9-CM codes 070.22, 070.23, 070.32, 070.33, V02.61) [16,23,24,28] diagnosed with CKD (ICD-9-CM code 585) [27,31], received renal transplantation (ICD-9-CM code V420), or were on dialysis therapy, defined as having a catastrophic illness registration card for ESRD (ICD-9-CM code 585) [32] from 1996 to the index date. All Taiwanese patients who develop ESRD and need long-term dialysis can apply for catastrophic illness registration cards from the Bureau of National Health Insurance, and have no copayments for health care. The diagnostic accuracy of ESRD was confirmed by both ICD-9 code 585 and inclusion in the Registry for Catastrophic Illness Patient Database, a subpart of the NHIRD [32]. HCV-infected subjects with the following pre-existing or co-existing comorbidities were excluded: diabetes (ICD-9-CM code 250), hypertension (ICD-9-CM codes 401–405), hyperlipidemia (ICD-9-CM codes 272–272.4), coronary heart disease (ICD-9-CM codes 410–414), and liver cirrhosis (ICD-9-CM codes 571.2, 571.5, 571.6). Previous research indicated that these conditions were associated with HCV through increased insulin resistance or oxidative stress [19,33]. Ultimately, 3182 subjects with newly identified HCV but none of these comorbidities were enrolled.

The HCV-uninfected control group was selected from the remaining subjects in the NHIRD. Subjects were excluded if they were diagnosed with HCV from 1996 to 2010. For each HCV-infected subject, 4 HCV-uninfected subjects were randomly selected. These uninfected subjects had matching gender, age, and index date (defined as the same year of the index date of the matched case). A total of 12728 HCV-uninfected control subjects were enrolled.

Subjects in both groups were tracked for 6 years (starting from the index date) to identify those who developed CKD but did not have catastrophic illness registration cards for ESRD starting renal replacement therapy. Cases were censored if they died or withdrew from the insurance program during the follow-up period. CKD is an important public health problem in Taiwan, so the CKD Prevention Committee of the Taiwan Society of Nephrology (TSN) launched a nationwide CKD Preventive Project in 2004 for the diagnosis and close supervision [34]. The TSN adopted the simplified Modification of Diet in Renal Disease (MDRD) equation in late 2005 for calculation of estimated glomerular filtration rate (eGFR) [34,35]. Diagnosis of CKD stages 1 to 5 was according to the National Kidney Foundation’s Kidney Disease Outcome Quality Initiative (KDOQI) [36], and used ICD-9 code 585 for national 5-stage CKD surveillance, the same as America [31].

For multivariate analysis, the association of the following factors with CKD were calculated: geographic region of residence (northern, central, southern, and eastern Taiwan) [1], urbanization level (urban, suburban, and rural) [27], and socioeconomic status [37]. Enrolee category (highest to lowest: EC1, EC2, EC3, and EC4) was used as a proxy for socioeconomic status [27]. The number of healthcare visits [25,30] in 1 year before study entry was also considered as a confounder.

### Statistical analysis

All data were analyzed using SAS version 9.2 (SAS Institute, Inc., Cary, N.C.). A two-sided *p*-value less than 0.05 was considered statistically significant. A *χ*^2^ test was used to compare categorical variables of the two groups, and the Kaplan-Meier method was used to estimate the cumulative risk of CKD during the 6-year follow-up. Person-years of follow-up were calculated for each subject from the index date to date of CKD diagnosis, date of death, or the end of the study (6-year follow-up), whichever occurred first. Incidence rate was calculated by dividing the number of CKD cases by the total person-years of follow-up. A Cox proportional hazard regression model was used to estimate hazard ratios (HRs) and 95% confidence intervals (CIs) after adjustment for covariates. The assumption of proportional hazards was confirmed by assessment of log minus log survival plots for patterns of nonproportionality (convergence, divergence, and crossing of curves) and the proportional assumption was satisfied.

## Results

### Patient characteristics

Table 1 shows the sociodemographic characteristics and comorbidities of the HCV-infected and HCV-uninfected control groups, who were matched for age and gender. Subjects in the HCV-infected group were more likely to reside in rural areas, live in central, eastern, or southern Taiwan, have lower socioeconomic status (EC2 or EC3), and have more healthcare visits (≧11) in 1 year before study entry (*p* < 0.0001 for each comparison). As expected, the HCV-uninfected control group had more comorbidities (diabetes, hypertension, coronary heart disease, and liver cirrhosis) because HCV-infected subjects with these comorbidities were excluded.

**Table 1:** Sociodemographic characteristics and comorbidities of the hepatitis C virus (HCV)-infected and HCV-uninfected control groups in Taiwan, 1998–2004 (n = 15910)

**Variables**	**HCV-infected group (n = 3182) N (%)**	**Control group (n = 12728) N (%)**	***p***
Gender			1.0000
Male	1619 (50.9)	6476 (50.9)	
Female	1563 (49.1)	6252 (49.1)	
Age (year)			1.0000
18–39	1319 (41.5)	5276 (41.5)	
40–49	874 (27.5)	3496 (27.5)	
50–59	552 (17.3)	2208 (17.3)	
60–69	315 (9.9)	1260 (9.9)	
≧70	122 (3.8)	488 (3.8)	
Diabetes			<0.0001
Yes	0 (0)	981 (7.7)	
No	3182 (100)	11747 (92.3)	
Hypertension			<0.0001
Yes	0 (0)	1726 (13.6)	
No	3182 (100)	11002 (86.4)	
Coronary heart disease			<0.0001
Yes	0 (0)	838 (6.6)	
No	3182 (100)	11890 (93.4)	
Hyperlipidemia			<0.0001
Yes	0 (0)	1031 (8.1)	
No	3182 (100)	11697 (91.9)	
Liver Cirrhosis			<0.0001
Yes	0 (0)	96 (0.8)	
No	3182 (100)	12632 (99.2)	
Geographic region			<0.0001
Northern	1017 (32.0)	6157 (48.4)	
Central	922 (29.0)	2916 (22.9)	
Eastern	81 (2.5)	292 (2.3)	
Southern	1162 (36.5)	3362 (26.4)	
Urbanization level			<0.0001
Urban	818 (25.7)	4076 (32.0)	
Suburban	1450 (45.6)	5891 (46.3)	
Rural	914 (28.7)	2760 (21.7)	
Enrollee category			<0.0001
1	1355 (42.6)	5978 (47.0)	
2	107 (3.4)	270 (2.1)	
3	1250 (39.3)	4367 (34.3)	
4	470 (14.8)	2113 (16.6)	
No. of health care visits in 1 year before study entry			<0.0001
<10	794 (25.0)	5199 (40.8)	
11-20	974 (30.6)	3761 (29.5)	
21-30	660 (20.7)	1977 (15.5)	
31-40	370 (11.6)	850 (6.7)	
≧41	384 (12.1)	941 (7.4)	

### Cox proportional hazard regression analysis

**Figure 2:**
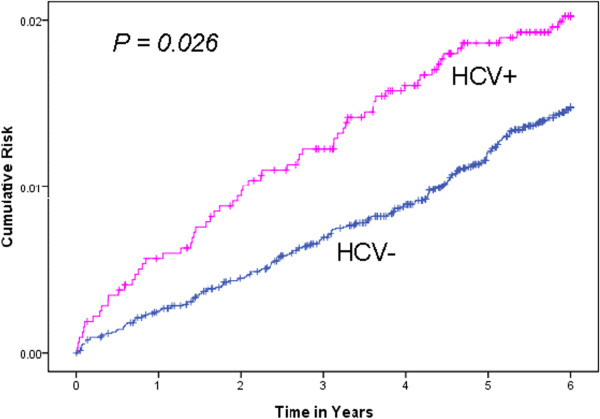
Cumulative risk of CKD in HCV-infected subjects and HCV-uninfected controls during a 6 year follow-up period.

Table 2 shows the distribution of CKD during the 6-year follow-up period for the two groups. The NHIRD tracked the use of all medical services by all subjects during the entire study period. The HCV-infected group had a mean follow-up duration of 5.88 years, and the control group had a mean follow-up duration of 5.92 years. At the end of follow-up, 64 subjects (2.0%) from the HCV-infected group and 187 subjects (1.5%) from the control group developed CKD. The incidence rate of CKD was significantly higher in the HCV-infected group than in the control group (3.42 *vs.* 2.48 per 1000 person-years, *p* = 0.02). The HCV-infected group also had a higher cumulative risk of CKD than the control group (Figure 2; *p* = 0.026). The mean time to diagnosis of CKD was less in the HCV-infected group than the control group (2.36 *vs.* 3.13 years, *p* = 0.003). Multivariate analysis with adjustment for age, gender, comorbidities, geographic region, urbanization level, enrolee category, and number of healthcare visits in 1 year before study entry indicated that the adjusted hazard ratio (aHR) for CKD during the 6-year follow-up period was greater in the HCV-infected group than in the control group (aHR: 1.75; 95% CI: 1.25-2.43, *p* = 0.0009). If the number of healthcare visits was not included in this multivariate analysis, the aHR for CKD was 1.97 (95% CI: 1.43-2.73, *p* < 0.0001).

**Table 2:** Incidence rate and hazard ratios for chronic kidney disease (CKD) in the hepatitis C virus (HCV)-infected and HCV-uninfected control groups during the 6-year follow-up period (n = 15910)

	**Control group**	**HCV-infected group**	***p***
	**(n = 12728) N (%)**	**(n = 3182) N (%)**	
No. of CKD	187 (1.5)	64 (2.0)	0.028
Mean follow-up (y)	5.92	5.88	0.0004
Total follow-up (person-year)	75371	18697	
Incidence rate^a^	2.48	3.42	0.02
Mean time to CKD (y)	3.13	2.36	0.003
Crude HR (95% CI)	1.00 (reference)	1.38 (1.04-1.83)	0.026
Adjusted HR (95% CI)^b^	1.00 (reference)	1.75 (1.25-2.43)^†^	0.0009

Abbreviations: *CI* confidence interval, *HR* hazard ratio.

^a^Per 1000 person-years.

^b^Adjusted for age, gender, diabetes, hypertension, coronary heart disease, hyperlipidemia, liver cirrhosis, geographic region, urbanization level, enrolee category, and number of healthcare visits in 1 year before study entry.

^†^Note: Adjusted HR with 95% CI of CKD in association with HCV was 1.97 (1.43-2.73, *p* < 0.0001) if the number of healthcare visits was not included in multivariate analysis.

### Cox proportional hazard regression analysis of subjects younger than 70 years old and without the above-mentioned comorbidities at baseline

Tsui et al. [10] demonstrated that HCV-infected patients younger than 70 years, but not older than 70 years, had increased risk for development of ESRD. Thus, we used a sensitivity test to analyze the association between HCV and CKD for the subjects younger than 70 years and without the above-mentioned comorbidities at baseline (Table 3). This analysis consisted of 3060 HCV-infected subjects and 9700 HCV-uninfected subjects. At the end of the follow-up, the HCV-infected group had a significantly higher incidence rate and aHR for CKD (aHR: 2.12, 95% CI: 1.44-3.13, *p* = 0.0002), and this aHR was slightly higher than that for all subjects (2.12 *vs.* 1.75).

**Table 3:** Incidence rate and hazard ratios for chronic kidney disease (CKD) in the hepatitis C virus (HCV)-infected and HCV-uninfected control groups younger than 70 years old and without comorbidities at baseline during the 6-year follow-up period (n = 12760)

	**Control group (n = 9700)**	**HCV-infected group**	***p***
	**N (%)**	**(n = 3060) N (%)**	
No. of CKD	58 (0.6)	56 (1.8)	<0.0001
Mean follow-up (y)	5.96	5.88	0.0001
Total follow-up (person-years)	57770	18004	
Incidence rate^a^	1.00	3.11	<0.0001
Crude HR (95% CI)	1.00 (reference)	3.09 (2.14-4.47)	<0.0001
Adjusted HR (95% CI)^b^	1.00 (reference)	2.12 (1.44-3.13)	0.0002

Abbreviations: *CI* confidence interval, *HR* hazard ratio.

^a^Per 1000 person-years.

^b^Adjusted for age, gender, geographic region, urbanization level, enrollee category, and number of health care visits in 1 year before study entry.

## Discussion

Many previous cohort studies have examined the association of HCV and CKD, but the results have been contradictory. The results of the present cohort study, which had a longer follow-up period (6 years) than these previous studies, indicate that HCV is a significant risk factor for CKD in the absence of traditional CKD risk factors, even after controlling for potential confounders.

Our study has a number of strengths that should be noted. First, the study population was taken from a large computerized database, and was representative of the population of Taiwan (rather than a specific region), so the results can be generalized to the entire population of Taiwan. Second, selection bias was not significant because we included subjects with newly identified HCV from 1998 to 2004 and we selected HCV-uninfected controls from a simple random sample of the insured general population. Third, recall bias was not significant because we identified traditional CKD risk factors from this database. Fourth, retrospective inclusion of HCV-infected subjects without coexisting traditional risk factors and retrospective exclusion of individuals with a previous history of CKD allowed us to exclusively study newly diagnosed cases of CKD. This provided a more reliable assessment of the relationship between HCV and CKD risk. Fifth, sensitivity analysis, which excluded subjects older than 70 years and without comorbidities at baseline, also indicated a positive association of HCV and CKD. Sixth, we also considered urbanization level and socioeconomic status to reduce environmental effects [29]. Seventh, in contrast to previous cohort studies [9-12,16], our multivariate analysis considered the number of healthcare visits to minimize detection bias, because HCV-positive patients might have been more closely followed and therefore received earlier diagnosis of CKD. Previous reports also used this method [25,30]. Our results indicated a decreased aHR after adjusting for the number of healthcare visits (aHR: 1.97, 95% CI: 1.43-2.73, *p* < 0.0001 *vs.* aHR: 1.75, 95% CI, 1.25-2.43, *p =* 0.0009). This indicates that clinicians should consider this as a potential confounding factor in future studies [30]. This information provides reassurance that HCV(+) is associated with an increased risk of CKD, and this association noted in our study is not merely due to an early detection of CKD in patients with HCV(+). Finally, the NHIRD is a large database that provides sufficient statistical power and good follow-up information for valid assessment of the relationship of HCV infection and CKD.

In the current study, the incidence rate of CKD in HCV-infected subjects was 0.9 per 1000 person-years higher than that in HCV-uninfected controls. This corresponds to approximately one additional case of CKD annually per 1000 HCV-infected subjects. Moreover, HCV-infected subjects without comorbidities were 1.75-fold more likely to develop CKD after adjustment for confounders, and to develop CKD nine months earlier than HCV-uninfected controls with comorbidities. These findings have important implications for prevention of CKD in Taiwan, where the prevalence of CKD is 11.9% [37] and the prevalence of HCV is 2-3% [38]. In particular, our results suggest that HCV-infected subjects should be routinely screened for CKD. However, future studies are needed to assess the effect of HCV eradication on the progression of CKD.

The risk of developing CKD in our HCV-infected subjects without traditional comorbidities (aHR: 1.75, 95% CI: 1.25-2.43) was higher than that in an American cohort of veterans (aHR: 1.3, 95% CI: 1.23-1.37) [9], a population that had higher prevalence of several CKD risk factors than our population. This difference may be related to differences in follow-up duration, comorbidities, and number of healthcare visits. However, our results are inconsistent with two other American cohort studies, one of an urban hospital [11] and another of a private health insurance population [2]. This difference may be attributed to the shorter follow-up durations of these two studies. Most kidney diseases have prolonged natural histories, so we believe that the strength and magnitude of the associations in our study are more valid because we used a 6 year follow-up period. In addition, two cross-sectional studies of a mostly female population in southern Taiwan [14,15], a region with a higher prevalence of HCV, also showed conflicting results, underlying the importance of the study population. A previous study of Asian HCV patients indicated that there were approximately equal numbers of males and females [39], consistent with our HCV cohort.

To date, there are two claim-based national cohort studies which employed ICD-9 coding that have validated an increased incidence and risk of developing ESRD in HCV(+) patients. A study of an American veteran population with male predominance [10] reported an incidence of 4.26 per 1000 person-years and an aHR of 1.68 (95% CI: 1.54-1.82). A study of the Taiwanese general population with similar sex ratio [16] reported an incidence of 2.36 per 1000 person-years and an aHR of 1.53 (95% CI: 1.17-2.01). The different incidences and aHRs for ESRD in these two studies may be due to the different gender ratios, because male CKD patients tend to experience more rapid deterioration of renal function than females [40]. There were some other differences in these two studies. Tsui et al. [10] validated that ESRD risk was independent of cirrhosis, but Su et al. [16] did not examine this issue. Su et al. validated a higher risk of ESRD in HCV(+) males than females, but Tsui et al. did not examine this. Tsui et al. indicated that HCV(+) patients had a lower prevalence of comorbidities at baseline than controls, and did not consider the Charlson comorbidity index (CCI) in the analysis, but Su et al. indicated that HCV(+) patients had a higher prevalence of comorbidities at baseline than controls and explicitly considered CCI in the analysis. Although CCI is an established method for summarizing the overall effect of comorbidities, it was developed for mortality analysis based on short-term outcomes of small samples of hospitalized patients, so should not be viewed as definitive for other types of studies [41]. In particular, Liu et al. [42] questioned whether the CCI accurately describes the comorbidity burden for ESRD and CKD patients and Schneeweiss et al. [43] concluded that comorbidity scores are unlikely to provide satisfactory confounder adjustments and do not standardize confounder adjustment across studies. Moreover, in addition to the present study, several well-known national claim-based studies in Taiwan [24,28,29] and elsewhere [2,33] that employed ICD-9 coding also did not adopt the CCI in their multivariate analyses. In the present study, subjects with traditional CKD risk factors were excluded from the HCV group but included in the control group. Thus, the number of comorbidities was significantly higher in the control group than the HCV group. This difference also strengthens our finding of a positive association between HCV and CKD. Our prior research validated this study design [27].

In the present study, the positive association between HCV and CKD in the absence of cirrhosis suggests that HCV may be directly responsible for renal injury. This result is consistent with a previous study which reported that a subset of HCV-infected subjects had renal involvement in the absence of cirrhosis [5]. The role of HCV in the pathogenesis of renal injury is uncertain, but we can suggest several possible mechanisms based on laboratory studies: *(i)* HCV may have direct cytopathic effects on renal parenchyma [44]; *(ii)* the patient’s systemic immune response to HCV infection is mediated by cryoglobulin, and this may lead to the formation of HCV-antibody immune complexes that disrupt renal function [44]; *(iii)* HCV may increase the expression of toll-like receptors in renal glomeruli [44]; and *(iv)* HCV may increase insulin resistance [18,19], leading to intrarenal overproduction of insulin-like growth factor-1 and transforming growth factor β, thus intensifying oxidative stress [20]. Some studies have also shown that the levels of inflammatory markers (interleukin 6, tumor necrosis factor α, highly sensitive C-reactive protein) are higher in HCV-infected patients than HCV-uninfected controls [19,33].

There are several limitations to this study. *First*, we did not consider CKD risk factors such as use of herbal supplements and analgesics and smoking because these data were not available in the NHIRD. Therefore, the association between HCV and CKD may be partially explained by residual confounding of these factors. However, previous research indicated that these factors contribute far less to CKD than traditional risk factors (aging, diabetes, and hypertension) [26], so their inclusion is unlikely to affect the results. *Second*, the diagnoses of HCV, CKD, and other comorbidities are based on ICD-9 codes, and misclassification is possible. However, many previous national cohort studies used ICD-9 codes for patients with chronic diseases and have validated this approach [1,2,16,23,24,26-30,33,45], so misclassification (if any) was likely to be random and thereby lead to underestimates of associations [25]. Furthermore, the NHI Bureau of Taiwan randomly reviews the charts and audits medical charges and imposes heavy penalties for inappropriate charges or malpractice. It is generally believed that these checks and balances ensure accurate coding [46] to minimize ascertainment bias. Claims data can be used to accurately identify patients with CKD because of a high positive predictive value (97.5%) [45]. Previous studies have documented the similarity of CKD diagnosis based on claims data of large administrative data sets and on eGFR [1]. Also, the prevalence of claims-identified CKD has risen substantially since 1999 because of increased recognition and coding of earlier-stage CKD [31]. Therefore, we believe that patients with 5-stage CKD were identified during the 6-year follow-up, through implementation of the nationwide CKD Preventive Project since 2004 and 5-stage CKD classification according to the simplified MDRD equation since late 2005. *Third*, patients with sub-clinical HCV infection may have been included in our HCV-uninfected controls. However, if HCV is causally associated with CKD, this misclassification would further strengthen our findings [16,28]. Moreover, the overall seroprevalence of anti-HCV in Taiwan has been estimated as 2 ~ 3% [38] and the prevalence of HCV in our cohort was within this range (2.1%). Therefore, the positive association between HCV and CKD in our cohort is relatively solid. *Fourth*, laboratory data (*e.g.* serum HCV RNA, creatinine levels, HCV genotype) is not available in the NHIRD. Thus, we could not consider the effects of CKD severity, viral count, or viral genotype. Notably, previous research indicates that ~78% HCV-infected patients have chronic viremia [47]. *Fifth*, our use of ICD-9 coding for diagnosis of HCV infection is likely to have excluded some subjects with mild disease, leading to severity bias that would bias away from the null hypothesis [16]. However, hepatitis is a major health problem and liver cancer is a leading cause of cancer deaths in Taiwan, so the government has developed rigorous guidelines for diagnosis [24] and has established programs for extensive community screening [16]. Hence, most HCV cases in Taiwan are asymptomatic and are identified through extensive screening programs rather than severe HCV cases diagnosed in hospitals. *Finally*, as with any observational study, potential unmeasured confounders may exist in cases and controls. However, we controlled for the confounding effect of medical attention by introducing the number of healthcare visits into the multivariate regression model [25]. Nonetheless, given the magnitude and statistical significance of the observed effects in this study, this limitation is unlikely to compromise our results.

## Conclusions

In summary, this nationwide 6-year follow-up cohort study indicates that there is a significant and independent association between HCV infection and CKD risk in the absence of traditional CKD risk factors. HCV-infected patients should be made aware of their increased risk for CKD and should be more carefully monitored for development of CKD. Further interventional studies are needed to evaluate the impact of HCV therapy on the progression of CKD.

## Competing interests

The authors declare that they have no competing interests.

## Authors’ contributions

Conceived and designed the experiments: YCC. Contributed reagents/materials/analysis tools: SKH, YCS. Performed the experiments: YCC. Analyzed the data: YCC, WYC, SKH, YCS. Wrote the paper: YCC. Provided constructive opinions and suggestions: YCC, WYC, SKH, YCS, SJH. Study supervision: SKH, YCS. All authors read and approved the final manuscript.

## Pre-publication history

The pre-publication history for this paper can be accessed here:

http://www.biomedcentral.com/1471-2369/14/187/prepub
